# DERT Science Retreat 2005 Report

**Published:** 2006-04

The 2005 NIEHS Division of Extramural Research and Training’s annual scientific retreat was titled “Practical and Potential Clinical and Public Health Outcomes Derived from Basic Environmental Health Sciences Research.” The retreat focused on integrating the science in our extramural research portfolio into the clinical and public health arena. Participants were challenged to think broadly and beyond their basic research foci and specific areas of expertise, identifying means to facilitate integration and interaction with clinicians and public health practitioners.

The first session dealt with clinical and public health research and began with an overview of the definitions of terms common to clinical and public health research. Invited speakers described the opportunities and challenges involved with human experimentation in the environmental sciences and the challenges of evaluating public health outcomes derived from basic environmental health science research.

The second session examined models of transition from basic to clinical and/or public health research. Speakers focused on three areas in which successful transitions have been achieved: the link between benzene exposure and leukemia; the effects of arsenic exposure on public health in a Bangladesh project studying the effects of drinking water contamination and the development of cancer; and the role of allergens on asthma.

The third session began with a group discussion on developing a more integrated approach toward science. This was followed by the formation of three breakout groups to discuss areas in which the NIEHS has substantial extramural investments: neurodegeneration, reproduction, and lung and cardiovascular disease. In each discussion, participants attempted to identify, within that area of science, opportunities for integration, means to facilitate the incorporation of integrative opportunities to extramural investigators, and measures of successful integration.

The final session consisted of readback presentations from the breakout panel discussions. Themes emerging from this session include the need to analyze the current portfolio to identify interactive, integrative opportunities; and the need to expand our research portfolios and increase awareness and interest in the environmental component of disease. Some specific recommendations indicated that the NIEHS should

- establish partnerships with professional societies, provide written commentaries in their scientific journals, and propose and plan special scientific sessions at society annual meetings;
- develop the next generation of investigators, perhaps through the development of institutional career awards, the establishment of integrated mentoring for postdocs, and the support of training for clinical fellows in environmental health research; and
- consider international research opportunities, as foreign populations exist that are exposed to environmental toxicants.

The general perception was that the research community would be receptive to our goal of integrating basic research with clinical and public health outcomes.

## Invited Speakers and Discussion Leaders

Wayne Cascio

East Carolina University

Bernard Goldstein

University of Pittsburgh

Joseph Graziano

Columbia University

Steven Kleeberger

NIEHS

Philip Landrigan

Mount Sinai School of Medicine

Stephanie London

NIEHS

Richard Mailman

University of North Carolina, Chapel Hill

Fernando Martinez

University of Arizona

Stephen Rappaport

University of North Carolina, Chapel Hill

Martyn Smith

University of California, Berkeley

David Walmer

Duke University

Paul Watkins

University of North Carolina, Chapel Hill

